# Orientational analysis of atomic pair correlations in nanocrystalline indium oxide thin films

**DOI:** 10.1107/S2052252523010357

**Published:** 2024-01-01

**Authors:** Justin M. Hoffman, Niklas B. Thompson, Olaf Borkiewicz, Xiang He, Samuel Amsterdam, Zhu-lin Xie, Aaron Taggart, Karen L. Mulfort, Alex B. F. Martinson, Lin X. Chen, Uta Ruett, David M. Tiede

**Affiliations:** a Chemical Sciences and Engineering, Argonne National Laboratory, 9700 South Cass Avenue, Lemont, IL 60439, USA; b X-ray Science, Argonne National Laboratory, 9700 South Cass Avenue, Lemont, IL 60439, USA; c Materials Science Divisions, Argonne National Laboratory, 9700 South Cass Avenue, Lemont, IL 60439, USA; UCL, United Kingdom

**Keywords:** orientations, conductive thin films, atomic layer deposition, grazing-incidence diffraction, pair distribution functions, materials science, total X-ray scattering, inorganic chemistry, film anisotropy, amorphous materials

## Abstract

The application of grazing-incidence total X-ray scattering (GITXS) for pair distribution function (PDF) analysis using >50 keV X-rays from synchrotron light sources has created new opportunities for structural characterization of supported thin films with high resolution. Compared with grazing-incidence wide-angle X-ray scattering, which is only useful for highly ordered materials, GITXS/PDFs expand such analysis to largely disordered or nanostructured materials by examining the atomic pair correlations dependent on the direction relative to the surface of the supporting substrate. A characterization of nanocrystalline In_2_O_3_-derived thin films is presented here with in-plane-isotropic and out-of-plane-anisotropic orientational ordering of the atomic structure, each synthesized using different techniques. The atomic orientations of such films are known to vary based on the synthetic conditions. Here, an azimuthal orientational analysis of these films using GITXS with a single incident angle is shown to resolve the markedly different orientations of the atomic structures with respect to the planar support and the different degrees of long-range order, and hence, the terminal surface chemistries. It is anticipated that orientational analysis of GITXS/PDF data will offer opportunities to extend structural analyses of thin films by providing a means to qualitatively determine the major atomic orientation within nanocrystalline and, eventually, non-crystalline films.

## Abstract 2

Grazing-incidence total X-ray scattering allows for orientational pair distribution function analysis of indium oxide thin films with only a single measurement. Elucidation of information such as effects of film anisotropy on bond lengths in specific orientations relative to the substrate and determination of orientation in X-ray amorphous films is demonstrated.

## 1. Introduction

Understanding the relationship between the atomic structure and macroscopic properties of interfacial thin films is a cross-cutting challenge for a broad range of applications, including artificial photosynthesis, heterogeneous catalysis and energy storage. For example, orientational atomic order and organ­ization play key roles in charge-carrier dynamics in thin films of electronically anisotropic materials and properties in optically anisotropic thin films (Wenk & Houtte, 2004 ▸; Nelson *et al.*, 2009 ▸; Xia *et al.*, 2014 ▸). Many thin films can be considered as quasi-2D polycrystalline or layered materials in which structured grains are unoriented in-plane but show variable extents of preferred out-of-plane orientation due to material-specific interactions between thin-film constituents and the supporting interface, especially for very thin films.

Correlations between anisotropy and functional characteristics of supported thin films have been investigated extensively using grazing-incidence wide-angle X-ray scattering (GIWAXS) (Rivnay *et al.*, 2012 ▸; Ye *et al.*, 2020 ▸). In this technique, a supported thin film is placed nearly parallel to the incoming X-ray beam, with an incidence angle near that of the critical angle, generally <1°. The scattered X-rays are recorded by a 2D detector positioned close to the sample, giving a 2D pattern of Bragg peaks or Debye–Scherrer rings with a *Q* range of ∼0.1–6 Å^−1^. However, the restricted *Q* range covered by GIWAXS measurements limits the use of direct Fourier transform methods for real-space structure characterization, which is necessary to examine the local and short-range ordering. As a result, real-space structural information is often restricted to inference based on measurements of azimuthal angle-dependent Bragg diffraction features that can be indexed to known structures. This approach has severe limitations for the characterization of films without well characterized Bragg diffraction.

Total scattering measured with high-energy X-ray radiation, typically >50 keV, and with high spatial resolution, *Q* > 20 Å^−1^, enables pair distribution function (PDF) analysis to achieve real-space structure characterization across distance scales that range from atom pair distances across individual bonds to the dimensions of nanoscale materials (Billinge, 2019 ▸; Billinge & Kanatzidis, 2004 ▸; Petkov, 2008 ▸; Egami & Billinge, 2012 ▸). With a *Q*_max_ of 20 Å^−1^, this gives a real-space resolution of 0.31 Å, allowing for precise measurement of atomic distances. While the energy and *Q* range stated here are not definitive limits for PDF analysis, achieving the highest *Q* range possible is desired for high-quality data, which is best achieved with high-energy X-rays. High-photon-energy total scattering capabilities have been extended to grazing-incidence total X-ray scattering (GITXS) for PDF analysis of supported inorganic thin films (Dippel *et al.*, 2019 ▸, 2020 ▸; Shyam *et al.*, 2016 ▸; Stone *et al.*, 2016 ▸; Roelsgaard *et al.*, 2019 ▸) and even indium oxide few-atom clusters within an organic polymer thin-film host matrix (He *et al.*, 2020 ▸). Furthermore, a computational framework for analysis of orientational texture in polycrystalline thin films has been developed, based on PDFs using multiple scans at a large range of incidence angles (Gong & Billinge, 2018 ▸), and experimental demonstrations have been achieved using both transmission (Harouna-Mayer *et al.*, 2022 ▸) and grazing incidence (Roelsgaard *et al.*, 2019 ▸) X-ray total scattering geometries. Additionally, several reports have shown orientational analysis for polycrystalline materials using transmission X-ray scattering, including the use of directional PDFs, similar to what is reported here (Egami *et al.*, 1995 ▸; Usher *et al.*, 2015 ▸; Suzuki *et al.*, 1987 ▸).

In this report, we present a qualitative orientational analysis of GITXS measurements for a series of four conductive indium oxide thin films supported on glass substrates that were prepared using different synthesis techniques. These samples are each nanocrystalline with grain sizes less than the thickness of each sample, as shown by Fig. S9 of the supporting information. Additionally, each film-growth technique used is well known to give highly uniform films. Each sample and corresponding background was measured at a single incident angle and collected with a 2D detector. After subtracting the background, this left a single 2D detector image that could be analyzed directly without the need for collecting additional images at different incident angles, as has been reported previously (Harouna-Mayer *et al.*, 2022 ▸). Analysis is carried out on single frames of GITXS 2D detector images. The samples included ∼200 nm thick crystalline commercial indium tin oxide (ITO) on glass, a 50 nm nanocrystalline ITO layer grown by atomic layer deposition (ALD) directly on glass (Emery *et al.*, 2016 ▸), a nanocrystalline In_2_O_3_ film grown by sequential infiltration synthesis (SIS) into a 100 nm thick poly(methyl methacrylate) film supported on glass (Waldman *et al.*, 2019 ▸; Taggart *et al.*, 2021 ▸) and a 50 nm amorphous indium zinc oxide (IZO) layer grown by ALD directly on glass (Sheng *et al.*, 2016 ▸). The conductive oxides are of particular interest as possible substrates for future *operando* photoelectrochemical GITXS measurements. The extent of out-of-plane orientational order for the domains – whether nanocrystalline or amorphous depending on the film – with respect to the planar support was analyzed by extracting azimuth-angle-resolved PDF patterns obtained from 2D GITXS detector images. Two-dimensional GITXS detector images were transformed to reciprocal-space spherical coordinates and corrected for grazing-incidence geometry. This approach follows from the methods widely used for the analysis of supported films by GIWAXS (Baker *et al.*, 2010 ▸), and is now extended to GITXS. The analysis shows that these indium oxides differ in their degree of long-range order and orientational alignment that result from different synthetic methods and the nature of the oxide–support interactions. Variations in the structural orientations may have implications on charge transport, surface chemistry and subsequent epitaxy.

## 2. Results and discussion

**Figure 1:**
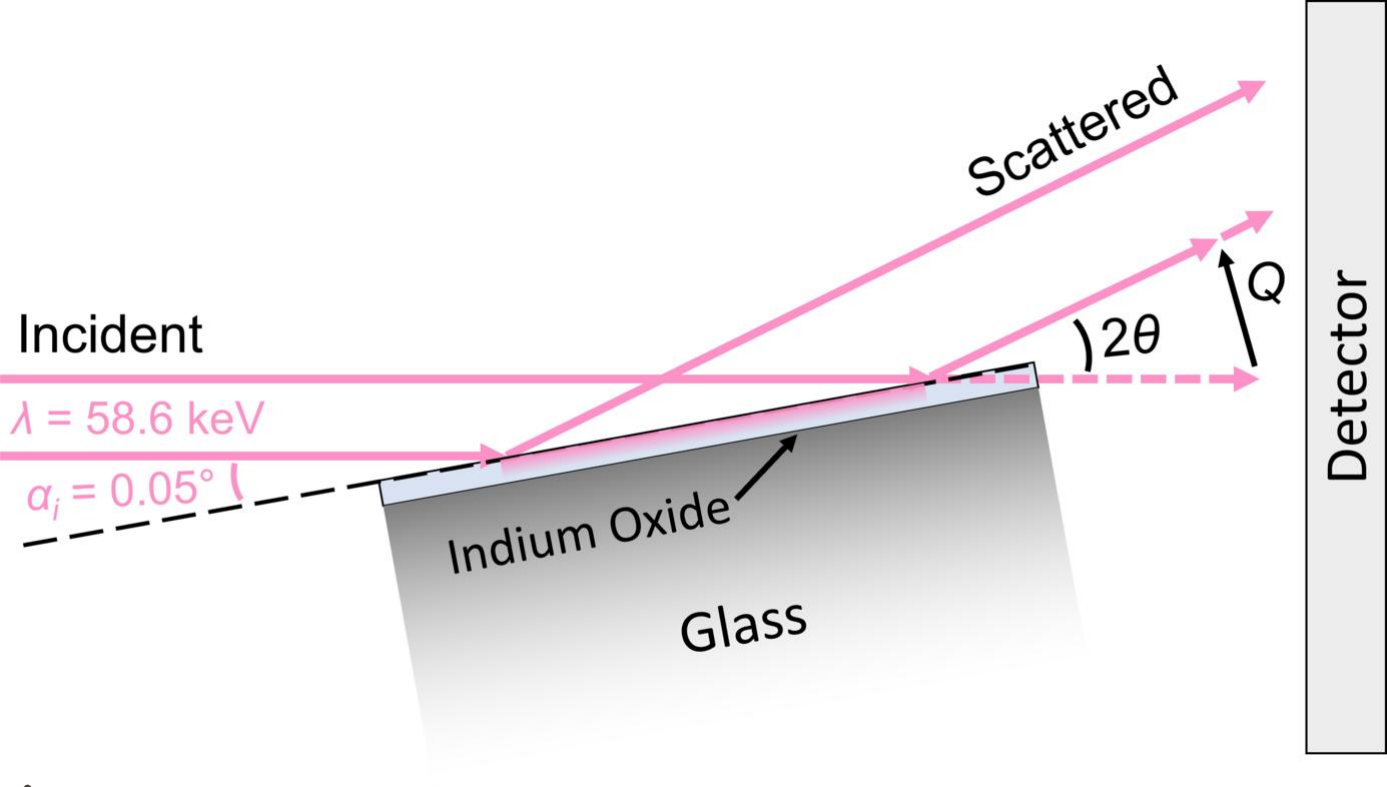
A schematic drawing of the geometry for the GITXS measurement of indium oxide thin films supported on planar glass supports. Neither the distances, thickness nor angles are to scale in order to allow simple visualization of the grazing-incidence geometry. The distance from the center of the sample to the detector was 220.0 cm for all samples except SIS-grown In_2_O_3_, which was 415.0 cm. The cross-section profile of 2–5 µm vertically focused incident and scattered X-ray beams are represented by magenta rays. The planar indium oxide/glass sample is positioned with an angle of incidence, α_i_. The scattered X-rays occur about an azimuth cone distributed around the incident beam, defined by the scatter angle, 2θ, and corresponding momentum-transfer vector **Q**, with magnitude *Q* = (4π/λ) sin θ, that is intercepted by a 2D detector. In this case, the azimuth cone is simplified into a scattering vector for clarity. The magenta shading schematically represents the X-ray footprint and penetrated volume.

The measurement geometry for GITXS characterization of the indium oxide thin films on planar supports is shown in Fig. 1 ▸. At beamline 11-ID-B at the Advanced Photon Source (APS), samples were mounted on a custom-made sample holder on a hexapod (PI-840) with angular precision of the order of 0.1 millidegree. A Kapton capillary with standard CeO_2_ powder centered on the mounting stage was used as the calibrant. A 58.6 keV X-ray incident beam (or 86.7 keV for SIS-grown In_2_O_3_) with an ∼2–5 µm vertically focused beam size is used to intercept the surface of the supported indium oxide films at a shallow incident angle, α_i_, that is adjusted to be approximately at or barely below the critical angle. The attenuation depth of the incident beam is a function of the angle of incidence, X-ray wavelength and elemental composition of the film. Calculations presented in the supporting information estimate the critical angle for the indium oxide films investigated in this study to be ∼0.05° with a theoretical penetration depth of 13 nm for an ideal sample. Tuning of the experimental parameters for GITXS makes it possible to preferentially interrogate the supported layer with relatively small contribution from the underlying support (here, glass) to the scattering signal. There was weak residual, but non-negligible, signal from the glass, which was subtracted using a secondary pristine glass film as a background. As noted previously (Dippel *et al.*, 2020 ▸), the possibility to tune the penetration depth in a GITXS experiment is a key advantage compared with experiments performed in transmission geometries.

For the highest data quality, data were collected in sets of six five-minute intervals and averaged using the Python package *pyFAI* (Kieffer *et al.*, 2020 ▸). Masking and calibration procedures were performed using *pyFAI* (Kieffer *et al.*, 2020 ▸). The distance from sample to detector was 220.0 mm for each sample, except the SIS-grown In_2_O_3_ sample, which was measured at a distance of 415 mm. The length of the sample in the direction of the X-ray beam is ∼2 cm, and with such low incident angles, the footprint of the beam is expected to be 6 mm for a beam height of 5 µm. This will lead to a noticeable broadening effect for the peaks in *Q* and *r*. Like for any grazing-incidence experiment, these effects must be accounted for when discussing material properties such as coherence length that affect the broadness of the peaks. Additionally, background subtraction will be much more difficult for PDF experiments such as this if the footprints for the substrate and background are different. In the studied cases, the background is quite weak relative to the sample, so this is less of an issue. However, for weakly diffracting or low-density samples, particular care must be taken during data collection to prevent improper background subtraction.

Following data acquisition, the 2D GITXS detector images were transformed into reciprocal-space polar plots corrected for grazing-incidence geometry (Baker *et al.*, 2010 ▸) using the Python package *pygix* (Dane *et al.*, 2021 ▸). This transformation maps scattering intensity at the detector (*x*, *y*) positions onto the in-plane, *Q_xy_*, and out-of-plane, *Q_z_*, components of the azimuthally distributed scattering vector **Q**, with magnitude *Q* = (4π/λ) sin θ, where 2θ is the scattering angle. In order for all the scattering to be collected and accurately shown in a single frame, the film must be isotropically ordered in the plane of the substrate, as is the case for the majority of thin films. For films with one or few domains and significant long-range order, the sample must be rotated along the axis normal to the substrate to create the isotropy required for single-measurement orientational analysis. Here, the high level of disorder in the plane of the nano-structured films means rotation was not required. All studied films were highly polycrystalline in the plane, like a 2D powder, but well ordered out of the plane of the substrate. Additionally, grazing-incidence measurements will miss the diffraction in the *Q_z_* direction, causing a distortion of the 2D diffraction pattern, notably leading to a ‘missing wedge’ along the vertical axis of the 2D data when 2D scattering patterns are transformed from angular to reciprocal space, as shown in Fig. S1, though the wedge is very narrow at high photon energies such that only data closest to the vertical axis are missing (Fig. S2) (Baker *et al.*, 2010 ▸). The energy used here leads to a small, but non-negligible, missing wedge, meaning that the data intensity near χ = 0° will be missing. In this data analysis, this corresponds to pair correlations that are normal to the substrate, meaning that these correlations will not be observed in a highly oriented sample. The transformed pattern has a radial axis of *Q* and an azimuthal axis of χ, where the χ axis is directly related to the orientational angle of structural features in the film to the supporting substrate. Here, χ = 0° is established to be along the *Q_z_* axis (the vertical axis) and χ = 90° is along the *Q_xy_* axis (the horizontal axis).

**Figure 2:**
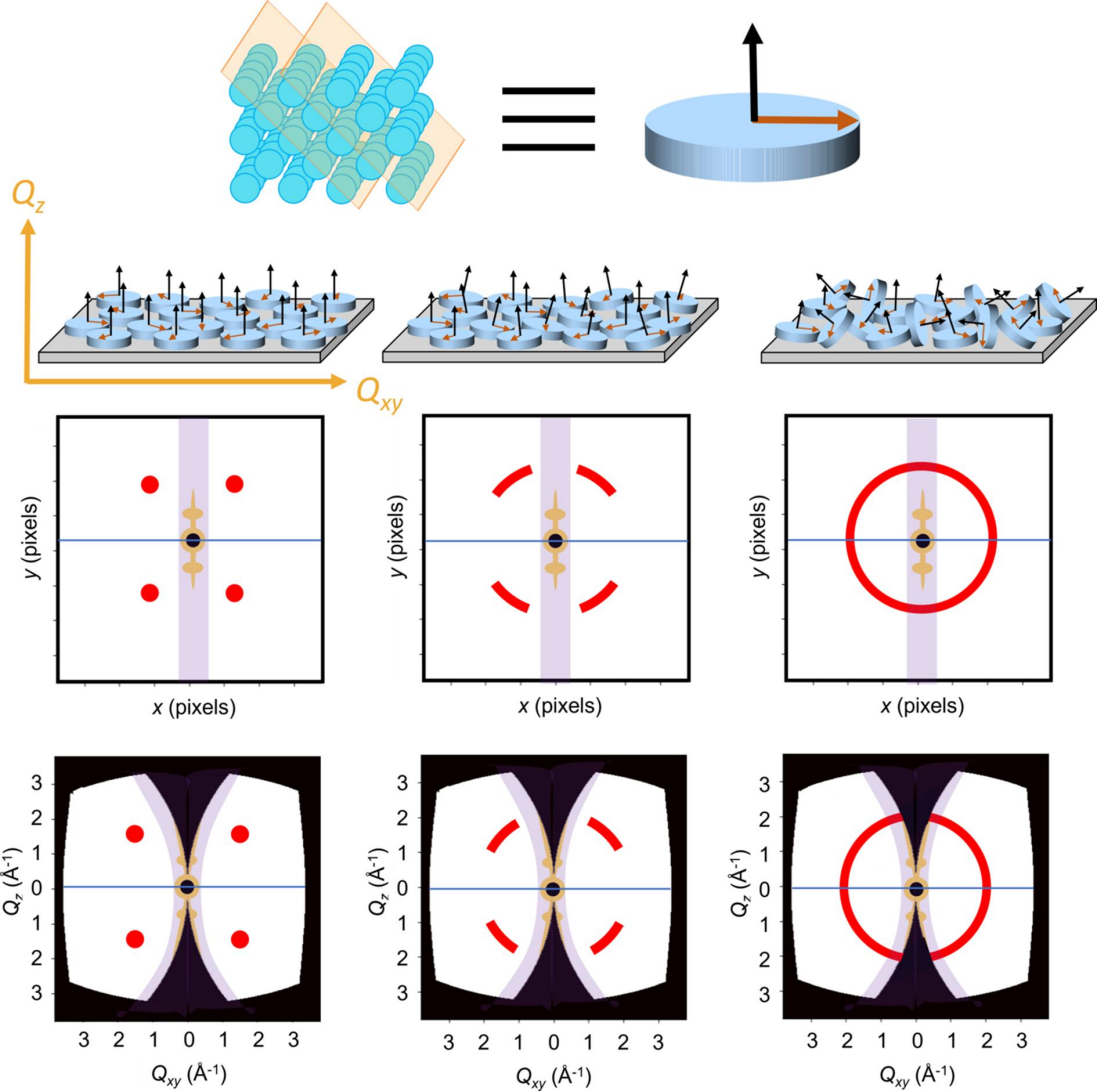
A diagram illustrating the effects of orientational disorder on GIXS detector images transformed to reciprocal-space coordinate frame *Q_x,y,z_* and corrected for grazing incidence. This is a general example that can be applied regardless of the *Q* range collected. An atomic model is shown in the top row. The blue circles represent atomic components and the orange planes represent Bragg planes. The distance between the planes is 3 Å in this example. This distance is the only distance used to generate the example patterns, and all other Bragg planes and atomic distances are ignored for the purposes of this example. Short cylinders are used as a simplified picture for different grains in the depicted atomic model. The black arrows on the cylinders are normal to the orange Bragg planes and the orange arrows are in the plane of the Bragg plane. Below this, polycrystalline films with different extents of orientational order are shown. The axes *Q_x_*, *Q_y_* and *Q_z_* are chosen based on the substrate, with the *Q_z_* axis normal to the substrate and *Q_x_* and *Q_y_* in the plane of the substrate. Because the indium oxide films are intrinsically isotropic along the plane of the substrate, the horizontal axis may be labeled *Q_xy_*. The films range from a highly oriented film on the left to films with increasing extents of orientational disorder about the *Q_xy_* direction on the right. Directly below the different film types, the expected detector images are shown with Bragg peaks or Debye–Scherrer rings resulting from the example Bragg plane in red. Intensity in yellow represents specular reflection, which is masked (purple rectangle). The horizon line is shown in blue. In the bottom row are the transformed GIXS patterns, showing that the peak is broadened along the azimuthal axis for less oriented films. The missing wedge along the *Q_z_* direction arises from the lack of intersection between the Ewald sphere and the detection plane (Baker *et al.*, 2010 ▸). While this missing wedge is essential for oriented films, for wholly isotropic films, it is unnecessary, though it is still shown for each film to be consistent.

There are several factors that must be accounted for when making these transformations. (1) This correction is only essential for oriented films, as films composed of 3D powders will not show any orientation effects, and this correction only transforms the data along χ, which should be equal for isotropic powders. For consistency’s sake, all films, regardless of orientation, will undergo this correction. However, the data may have inflated intensity near χ = 0° based on the orientational ordering of the film (Page *et al.*, 2014 ▸). (2) If the conditions required for specular reflection are satisfied, X-rays will reflect off the sample and manifest as intensity peaks along χ = 0° (Zabel, 1994 ▸). (3) An intense streak of intensity along χ = 0°, known as the Yoneda peak, is frequently seen as well and is the most significant when measurement is carried out at the critical angle of the material of interest (Yoneda, 1963 ▸). Each of these three factors results in data along χ = 0°, which occurs through different mechanisms than the scattering analyzed here and is thus not related to the corrections made by the geometric transformation resulting in the missing wedge. These data, if transformed, are no longer in the correct position, resulting in inaccuracies. While these data hold real value for other analysis, the orientational analysis presented here does not use this information. Because PDF analysis considers all intensity, these peaks are especially problematic, so this region should be excluded for this particular analysis, as demonstrated by the purple mask in Fig. 2 ▸.

The effect of orientational disorder and the arc of the Debye–Scherrer rings are illustrated for general grazing-incidence X-ray scattering (GIXS) in Fig. 2 ▸. Bragg peaks correspond to planes of atoms in a crystal structure, and there is a direct correlation of the angle χ on the detector and the angle between the Bragg planes and the substrate. So, if a Bragg peak is found at χ = 20°, the corresponding plane of atoms is at a 20° angle relative to the substrate, as demonstrated in Fig. 2 ▸. In GIWAXS experiments with *Q* < 6 Å^−1^, the detection of the angle dependence for the Bragg planes allows the orientation axis for known crystal phases to be resolved. Significantly, for GITXS measurements with *Q* > 18 Å^−1^, the pole plot transformation of the scattering patterns allows azimuth-angle-resolved PDF patterns to be obtained by integrating selected ‘slices’ of the 2D total X-ray scattering data representing different angular orientations in the film and performing PDF analysis on each slice. The result yields information beyond Bragg plane analysis by resolving how atomic pair correlations are orientated relative to the substrate.

However, care must be taken when quantifying this information, so it is necessary to define some limits when using this technique. In PDFs, the relative intensities of the peaks correspond to the scattering intensity of the atoms and the relative number of pair correlations, but accurately integrating different peaks to quantify the ratio of pair correlations is difficult because of a non-trivial non-zero background that is related to the density of the material. This background is not necessarily precisely the same for many thin films, such as films with grains larger in diameter than the film thickness, which would lead to differences in coherence length for different orientations. Additionally, if the substrate is orientated and not perfectly subtracted, spurious peaks will confound the data, which will vary in χ. Another factor to consider is in spatially non-uniform films, as the footprint may be of the order of millimetres to centimetres in length depending on the beam focus height, meaning that the region furthest from the X-ray source is slightly shifted to lower *Q* than the region furthest from the source. If the two regions have different anisotropy (or compositions), this will lead to a very complex pattern. For ultrathin films, poorly diffracting films, non-uniform films or films where a high degree of precision is required, further efforts in modeling the background, peak shifting and peak broadness in each arc cut are necessary. However, for the purposes of this article, a more qualitative analysis can be carried out, which is achievable for well oriented, crystalline and, relative to the substrate, highly scattering films. Here, the relative size of the PDF peaks in each slice can be compared with the same ratios in other slices to understand how the peaks are changing in intensity. The limits of this analysis for each film can then be discussed to show what conclusions this technique can and cannot provide. This type of analysis is described in detail for multiple In_2_O_3_-based films below.

Orientational GITXS analysis was first performed on commercial glass-supported nanocrystalline ITO films. The ITO structure is well understood and can be grown as films in a number of orientations, making it a good candidate to serve as a benchmark for orientational analysis (Emery *et al.*, 2016 ▸). ITO crystallizes in a bixbyite-like In_2_O_3_ structure, which can be imagined as an array of face-centered In(III) atoms with oxygen atoms filling ¾ of the tetrahedral vacancies (Nadaud *et al.*, 1998 ▸). In this structure, the In(III) atoms are cubically coordinated to six oxygen atoms and two vacancies, resulting in pseudo-octahedral InO_6_ units. These InO_6_ units are connected either through edge-sharing or corner-sharing bonding motifs. In ITO, the In(III) sites are partially substituted by Sn(IV) atoms and are charge balanced by additional oxygen atoms that fill up an equal number of these vacancies (Nadaud *et al.*, 1998 ▸). The ITO structure has higher coordination numbers overall as the vacancies are partially filled, but the bixbyite structure is generally preserved and will be used to describe the structure going forward.

**Figure 3:**
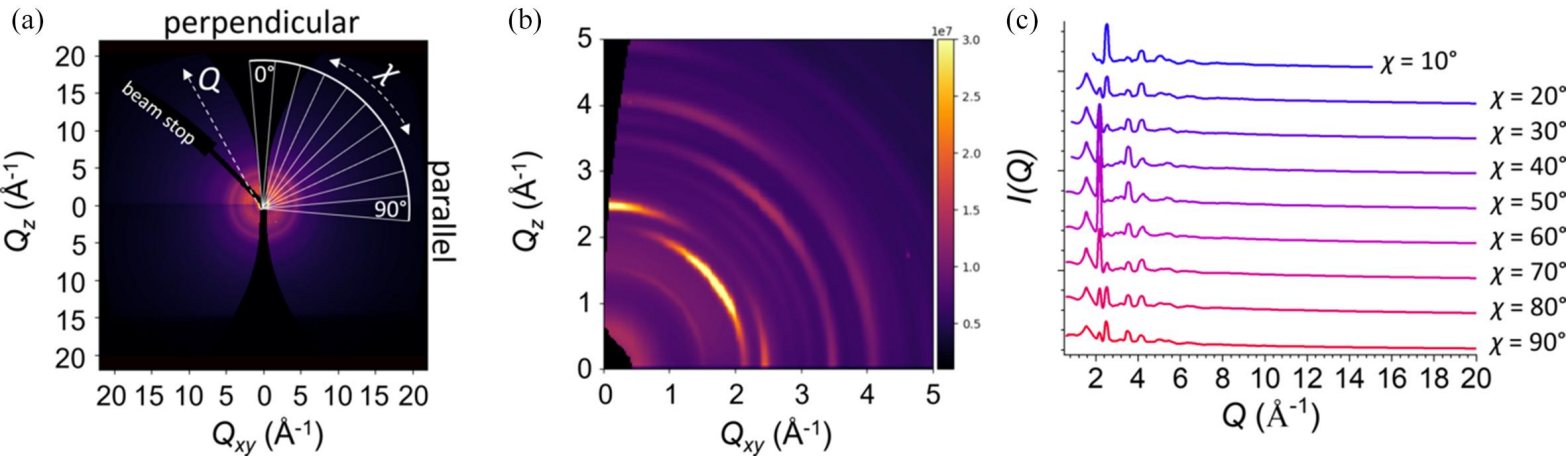
GITXS data analysis for commercial ITO on glass. Parts (*a*) and (*b*) show a reciprocal-space GITXS detector image, with (*b*) showing the region with *Q* ≤ 5 Å^−1^. The polar coordinates, *Q* and χ, are shown as dashed lines. Between the χ = 0° and χ = 90° directions, additional lines mark 10° slices along χ, based on the center of the slice. (*c*) The integrated slices from (*a*) are shown after the background subtraction of a glass substrate. The integrated data for the 10° slice are shorter due to the missing wedge and masked data, and will not be used for further analysis. The 90° wedge is shown here, but the data will not be used for further analysis because it is corrupted by effects at the horizon.

Reciprocal-space detector images for the commercial ITO sample show Bragg reflections spread out into arcs broadened along the azimuthal angle χ, indicative of a sample with a distribution of orientations centered around a primary orientation, Figs. 3 ▸(*a*) and 3 ▸(*b*). Azimuthal angle-resolved *I*(*Q*, χ) patterns were obtained by integrating in 10° slices centered at intervals of 10° from χ = 0° (along the vertical axis) to χ = 90° (along the horizontal axis) using the *pygix* Python package (Dane *et al.*, 2021 ▸), indicated in Fig. 3 ▸(*a*). Because of the missing wedge along the vertical axis, the data centered at 0 and 10° were not used. Additionally, the data centered at χ = 90° were excluded because of the shadowing along the sample horizon at low *Q_z_* due to absorption and refraction. For each integrated slice of the glass-supported ITO, corresponding χ-resolved *I*(*Q*, χ) slices from bare aluminoborosilicate glass surfaces (Delta Technologies, LTD, CB-1511) were subtracted to yield background-subtracted *I*(*Q*, χ) patterns for each slice in ITO, Fig. 3 ▸(*c*). Because separate substrates were used for the backgrounds and samples, the backgrounds for each sample were scaled accordingly, though the same scale was used for each azimuthal angle within each sample.

**Figure 4:**
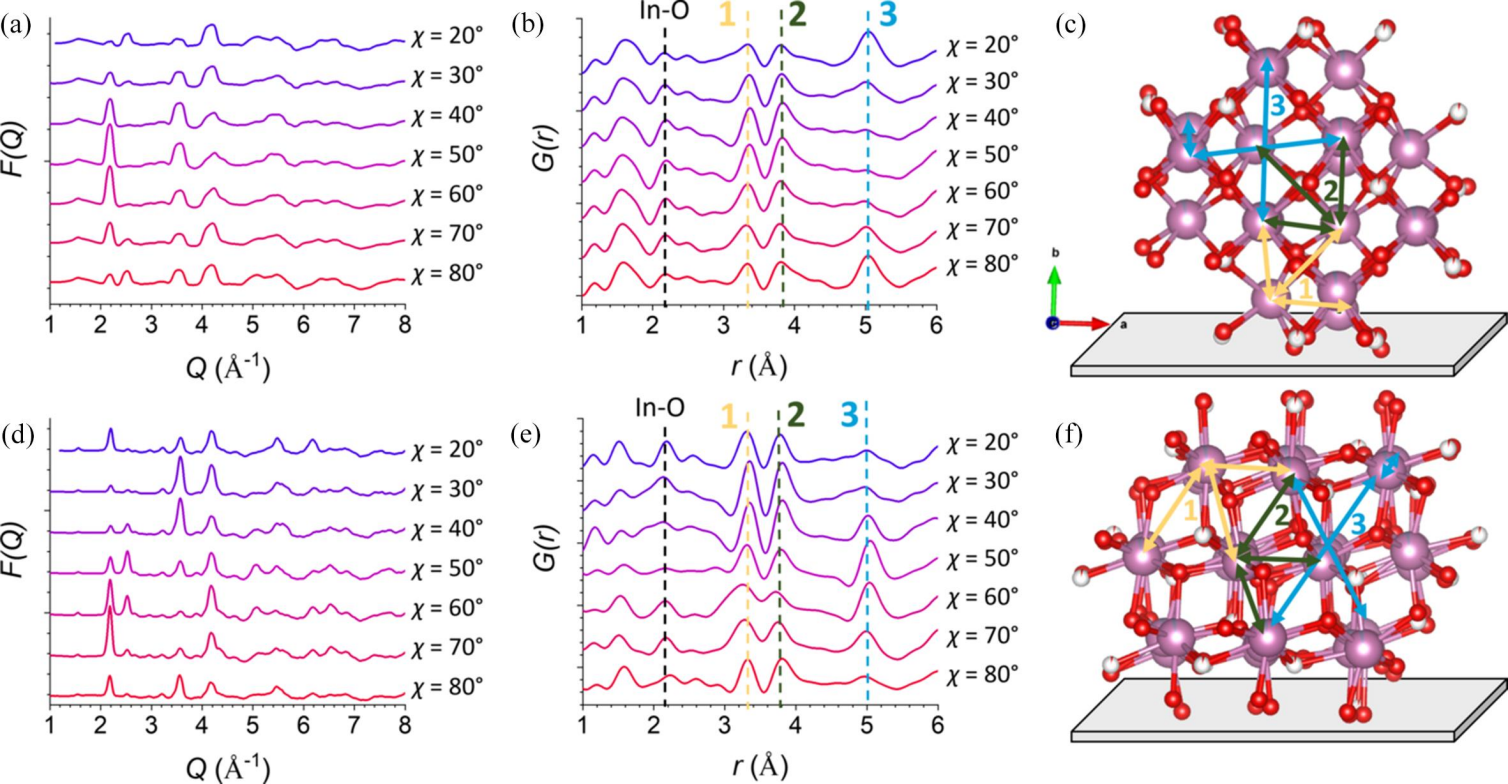
(*a*) *F*(*Q*, χ) for the commercial ITO film, shown for each of the integrated slices. (*b*) *G*(*r*, χ) for the commercial ITO film. (*c*) The proposed orientation of the ITO film with atomic pair correlations labeled corresponding to the peaks in (*b*). (*d*), (*e*), (*f*) *F*(*Q*, χ), *G*(*r*, χ) and proposed orientation for the ALD-grown ITO film, respectively. For (*c*) and (*f*): the gray plate indicates the glass substrate and the crystal structure is In_2_O_3_ (ICSD entry 14387, space group 206). The figures are generated with *VESTA* (Momma & Izumi, 2011 ▸).

The data were then converted to the angle-resolved reduced total scattering structure function *F*(*Q*, χ) for each angle using *PDFgetX3* (Juhás *et al.*, 2013 ▸). The resulting angle-resolved *F*(*Q*, χ) plots are shown for a portion of the *Q* range and the complete *Q* range measured in Figs. 4 ▸(*a*) and S3(*a*), respectively. Particularly for the *Q* range plotted in Fig. 4 ▸(*a*), the *F*(*Q*, χ) plots show angle-resolved variation of the shape peaks, such as those at 2.2 and 2.5 Å^−1^, which correspond to oriented diffraction from the (222) and (040) Bragg planes, respectively. Furthermore, the full *Q* range *F*(*Q*, χ), Fig. S3(*a*), can undergo Fourier transformation to yield azimuth-angle-dependent PDF patterns, *G*(*r*, χ), with sufficient *r*-space resolution for analysis and with resolution in χ for orientational analysis of specific atom pair correlations, Figs. 4 ▸(*b*) and S3(*b*). This is not achievable by analysis of selected Bragg peaks. In this case, specific peaks correspond to atomic pair distances associated with the crystal structure with orientation resolution. To give a general example, if there is a peak at *r* = 4 Å for a PDF pattern where χ = 40°, this would correspond to two atoms that are 4 Å apart. A line can then be drawn through both atoms in the structure, which would be at a 40° angle relative to the underlying substrate. Comparing this information with a known crystal structure allows for real-space orientational analysis. The structure is shown in Fig. S10 with simulated PDF patterns, which may be used as a reference for the fully isotropic case without distortions that may be present using the grazing-incidence geometry.

Looking now at the experimental *F*(*Q*, χ) and *G*(*r*, χ) data for the commercial ITO film, Figs. 4 ▸(*a*) and 4 ▸(*b*), respectively, the patterns are seen to vary for the different azimuthal slices. Overall, the peaks above 2 Å in the experimental *G*(*r*, χ) data match the positions for the peaks in the simulated isotropic pattern in Fig. S10, indicating that the atomic structure of the majority phase present in the film is In_2_O_3_. However, the differences in the peak intensities between each slice could indicate that the structure is oriented in the film. Looking at each of the peaks may reveal what the majority orientation is in the film. Considering the azimuthal dependence of the intensity of several of the low-*r* peaks will show how this methodology can be applied and show the limits of this technique to give qualitative information.

First, the peak at 2.2 Å, corresponding to the first-shell In—O bond length, is strongest for an orientation at 40–50° to the surface, indicating that the In—O bond directions are primarily diagonal to the substrate. Looking at the structure of ITO and remembering that all the In(III) sites are cubically coordinated, the structure would be oriented in the way shown in Fig. 4 ▸(*c*) to keep the In—O bonds at ∼45° angles to the substrate. However, peaks at lower *r* are difficult to use to draw conclusions. In particular, the weak scattering of oxygen compared with indium leads to a very weak first-shell In—O peak, which is further convoluted by additional ripples at low *r* that are common in PDFs and especially strong here, probably due to lower integrated signal from using only a 10° slice. Additionally, a peak around 1.5 Å, possibly corresponding to the silicon–oxygen bond in glass caused by imperfect background subtraction, is present in these data. These data were collected using separate substrates for the sample and background, and any aberration in substrate structure would easily cause such imperfect subtraction. This attests to the need to use either very precisely cut and aligned samples or samples grown *in situ* with the same substrate. Considering all of these factors, analysis of further higher-intensity peaks is needed to draw any clear conclusions.

Next, two peaks at distances 3.3 and 3.8 Å, labeled peaks 1 and 2 in Fig. 4 ▸(*b*), can be analyzed. The intensity of these two peaks in each azimuthal slice seems to vary a little, with the highest intensity occurring around 40–50°, though it is difficult to confidently state that any orientation can be definitely seen based on any apparent trend here. Firstly, the differences between the slices are weak enough to be possibly caused by imperfect subtractions. Additionally, as discussed above, this methodology relies on differences in the relative intensities of different peaks, so even if the peaks appear stronger in one slice, this is meaningless without a reference. Finally, looking at the structure itself reveals that these two peaks are not useful for orientational analysis. From the oriented bixbyite structure, Fig. 4 ▸(*c*), these correlations are seen to correspond to In–In distances between either edge-sharing (1) or corner-sharing (2) InO_6_ units. In both cases, there are multiple pair correlation directions corresponding to the In–In directions, all oriented in different directions in 3D space to one another. Thus, even if the structure was highly oriented, these pair correlation directions would be present at many different angles relative to the substrate. Because of this, these peaks are not useful for orientational analysis unless much shorter azimuthal step sizes are used and the film has a sufficiently narrow range of orientation distributions.

Another major peak can be used to complete this orientational analysis. The peak at 5.0 Å (3) is dependent on χ, with very strong intensity corresponding to bond vectors parallel and perpendicular to the substrate and almost no intensity corresponding to bond vectors diagonal (40–60°) to the substrate. This peak corresponds to another In–In distance, with three corresponding vectors in the crystal structure that intersect at ∼90° angles. Based on the orientational PDF data, these vectors must be oriented only parallel and perpendicular to the substrate. Because of the cubic symmetry, it does not matter which vectors are chosen; they are all crystallographically equivalent. This result is in agreement with the structural orientation shown in Fig. 4 ▸(*c*), which also has the In—O bonds diagonal to the substrate and the In–In (1) and (2) vectors spread out at three different orientations, *i.e.* the film grows mainly with the (010) direction of the In_2_O_3_ structure perpendicular to the substrate while isotropic in the plane of the substrate due to the nanocrystalline nature of the film. This orientation analysis is highly intuitive, giving qualitative information on the major orientation in the film with the requisite of a known crystal structure. The results also match that of the traditional analysis based on Bragg peak position (Fig. S5). Additional In—O peak intensities at *r* = 2.2 Å are seen at all angles, implying that some of the sample is un­oriented. This is not as obvious from the GIWAXS data, which are not amenable to analysis of short-range order.

In contrast to the commercial ITO film on glass, we found that another nanocrystalline ITO film, this time grown by ALD directly onto glass under the process conditions selected for this work as described in the supporting information, exhibited a different orientation-resolved PDF pattern. These films were grown by depositing ten layers of InO_*x*_, then one layer of SnO_*x*_, and then alternating every 19 layers of InO_*x*_ with one layer of SnO_*x*_, ending with nine layers of InO_*x*_. This process was repeated 25 times to give a film thickness of ∼50 nm. The film was then annealed in the presence of hydrogen gas at 250°C to increase conductivity (Galazka *et al.*, 2013 ▸).

Following the same procedures as used for the commercial ITO, angle-resolved *F*(*Q*, χ) and PDF patterns were obtained for the ALD ITO, and are shown plotted in Figs. 4 ▸(*d*) and 4 ▸(*e*), respectively. In contrast to the commercial ITO glass sample, the first-shell In—O peak is detected with mostly uniform intensity at each χ angle. While the peaks do seem to slightly vary in intensity and position with varying χ, any changes are not substantially above the background noise to draw conclusions. The In–In (1) and (2) pair distance peaks are likewise similar intensity at all χ, which is again attributed to the multiple pair correlation directions in the crystal structure. However, the In–In (3) peak is most intense for diagonal orientations and has more intensity at the parallel orientation than at the perpendicular orientation. Based on this, a different orientation is proposed in Fig. 4 ▸(*f*) with the In—O pair correlations oriented in all directions and the In–In (3) peak oriented at a 50° angle to the substrate, suggesting that the film grows so that the (111) direction is perpendicular to the substrate surface. Because there is some intensity of the In–In (3) peak perpendicular and parallel to the substrate, it is likely there is again a distribution of crystallite orientations present in the film, with possibly the orientation shown in Fig. 4 ▸(*f*) representing a majority contribution. The film was also measured at its critical angle of 0.048° (Fig. S4). Because the attenuation of the X-rays into the film increases with incident angle, as described by equation (2) in the supporting information, the X-rays at the critical angle probe deeper into the bulk of the film than the results above. In this case, the patterns show broadening of the Bragg peaks along χ. This may suggest that different orientations are present at the glass/ITO interface, which is not probed at shallower incident angles. While interesting, this type of depth-dependent analysis in GITXS/PDFs is still being developed and will not be discussed further in this study.

Next, GITXS/PDF patterns for these crystalline thin films are compared with two indium oxide thin films with presumably less structural orientation: crystalline indium oxide grown by SIS (Waldman *et al.*, 2019 ▸) and IZO that is amorphous based on a lack of Bragg peaks in the X-ray scattering patterns, which agrees with previous IZO films grown with the same methodology (Sheng *et al.*, 2016 ▸).

**Figure 5:**
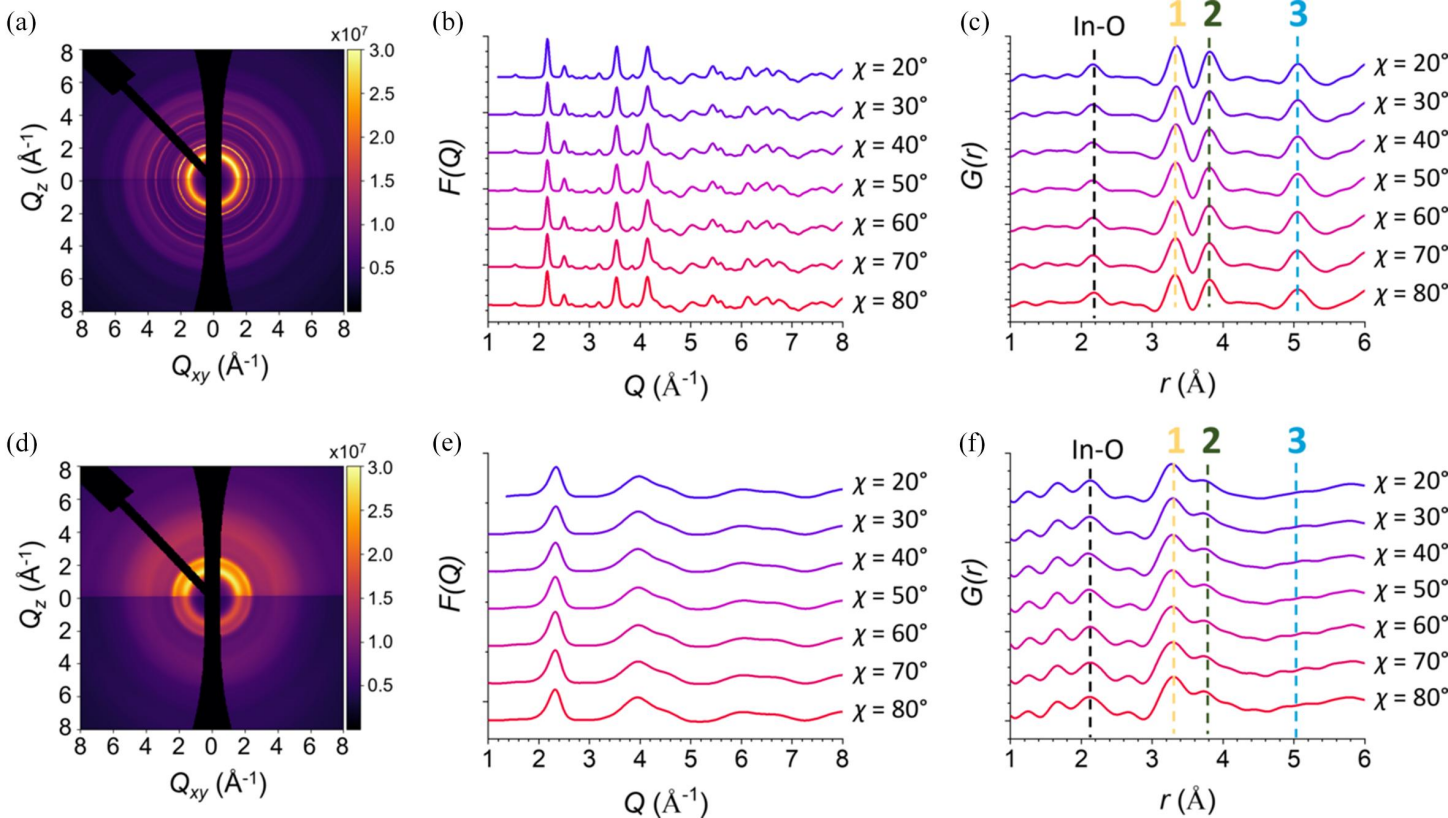
(*a*)–(*c*) The geometrically corrected 2D data, *F*(*Q*, χ) and *G*(*r*, χ) for the SIS-grown In_2_O_3_ unoriented film. (*d*)–(*f*) The geometrically corrected 2D data, *F*(*Q*, χ) and *G*(*r*, χ) for the ALD-grown IZO film. The labels for the peaks in (*c*) and (*f*) are at the same *Q* as for the ITO pair correlations in Fig. 4 ▸.

First, the nanocrystalline In_2_O_3_ film made using SIS (Waldman *et al.*, 2019 ▸; Taggart *et al.*, 2021 ▸) was analyzed. The process is performed by depositing indium oxyhydroxide clusters into a polymer matrix; in this case, poly(methyl methacrylate) (He *et al.*, 2020 ▸). Because the indium oxyhydroxide nucleates within an unoriented polymer thin-film matrix, the resulting film is probably unoriented as well. It is expected that the film will remain unoriented following annealing treatments to remove the polymer phase and dehydrate the soft oxyhydroxide to indium oxide, thereby increasing the long-range order. As seen in Fig. 5 ▸(*a*), the geometrically corrected 2D data appear as narrow Debye–Scherrer rings indicative of an unoriented polycrystalline film. Integrating different slices of the data as done above gives *F*(*Q*, χ) and *G*(*r*, χ) patterns that are almost identical for all slices, as shown in Figs. 5 ▸(*b*) and 5 ▸(*c*), respectively. In fact, comparing with the simulated isotropic PDF patterns in Fig. S10, each of the slices agrees well with the simulated data, a strong argument that the structure is isotropic in the film as well. This case is a good control example to show that an unoriented film does not possess any differences in peak intensities for different orientations. The full range of the data is shown in Fig. S7.

In the final example of GITXS/PDF analysis, the total scattering from an amorphous 50 nm IZO film deposited by ALD onto a planar glass substrate is shown, this time with α_i_ = 0.047°. Like the ALD ITO film, this film was deposited one sub-monolayer at a time, but, in this case, the zinc oxide was deposited every tenth ALD cycle starting after the fourth indium oxide cycle. This film differs structurally compared with the other films presented due to a loss of long-range order, which has been discussed elsewhere (Eguchi *et al.*, 2010 ▸). While the exact composition of the film here is beyond the scope of this article, considerations of the stoichiometry of the film will be needed for more quantitative analysis. For this film, the geometrically corrected 2D data in Fig. 5 ▸(*d*) have no narrow Debye–Scherrer rings or Bragg reflections, they only have broad Debye–Scherrer rings characteristic of an amorphous oxide. Because this technique deposits layers of indium and zinc sequentially, one may surmise that the structure is anisotropic, with different pair correlations occurring normal to the substrate in the direction of the deposition when compared with pair correlation within the same deposition layers within the plane of the substrate. To assess how well this methodology may or may not provide answers to such questions, the same orientational analysis can be applied to see if any differences are significant enough for qualitative analysis. The azimuthal angle-resolved *F*(*Q*, χ) patterns show only extremely subtle line-shape changes not discernable by eye, Fig. 5 ▸(*e*). While the corresponding *G*(*r*, χ) patterns in Fig. 5 ▸(*f*) do show weak angle-dependent variation in line shape and peak position for the first-shell In—O peak, the variations are once again very weak. In fact, these variations are even weaker than the changes in In—O peak intensities that were determined to be convoluted with spurious ripples in the crystalline indium oxide analysis above. This may be because there is no orientation of the film, but this methodology is simply not yet sensitive enough to make such conclusions. Further efforts must be made in improving the sensitivity of these measurements and improving data reduction with this more complex geometry for this type of analysis to apply to amorphous materials.

## 3. Conclusions

Orientational GITXS/PDF analysis of total X-ray scattering data taken in grazing-incidence geometry provides qualitative information on the orientational ordering of atomic pair correlations in thin films, which complements well established GIWAXS analysis of Bragg reflections in reciprocal space. Specifically, determining the presence of amorphous phases can be done more directly using the presence of emergent pair correlation peaks, which also has potential for structural characterization of minority amorphous phases. Unlike GIWAXS, GITXS/PDFs give direct structural information about the distances between atoms. By simply integrating the 2D data as azimuthal slices along χ, one can extract orientation-dependent pair correlations in the thin film. Even with a single measurement, the orientational data can be extracted, allowing for potential *in situ* studies and rapid collection of multiple films. These data can be used to determine the orientation of thin films in a very intuitive manner since the resulting histogram of pair correlations are real distances between atoms, which can be compared with either known structures or a proposed model. This is expected to be useful for quick orientational analysis during a GITXS/PDF measurement when further quantification is not needed, and it can provide information on systems that are inaccessible through traditional GIWAXS. An example would be for small nanoplates, which may align parallel to the substrate and be too small to give rise to Bragg peaks. With further technique development, we expect that reliable quantitative analysis is achievable and will be the focus of future studies. For example, by growing the films *in situ* to allow use of the same substrate, this will allow for dependable background subtractions, which will remove spurious peaks as well. Additionally, calculating the expected signal intensity variance along χ in real-space PDF patterns will allow for simulating 2D GITXS patterns, which can be fit to experimental data. Orientational analysis using GITXS/PDFs has been shown here to be useful for understanding the orientation of crystalline phases in a thin film using real-space analysis, and has potential to expand the analysis to amorphous phases and for studying fine-detailed anisotropy not accessible otherwise. We expect the method­ology outlined in this article to be the first step in developing orientational analysis using GITXS/PDFs as a standard characterization tool for complex thin films.

## Supplementary Material

Supporting information. DOI: 10.1107/S2052252523010357/ro5037sup1.pdf
