# Supplementary material for: Orientational analysis of atomic pair correlations in nanocrystalline indium oxide thin films
Source: IUCrJ. 2024 Jan 1;11(Pt 1):120–8. doi: 10.1107/S2052252523010357 (PMC10833382; doi:10.1107/S2052252523010357)
Supplement: Supplementary file 1 [file m-11-00120-sup1.pdf]

IUCrJ

Volume 11 (2024)

Supporting information for article:

Orientational analysis of atomic pair correlations in nanocrystalline indium oxide thin films

Justin M. Hoffman, Niklas B. Thompson, Olaf Borkiewicz, Xiang He, Samuel Amsterdam, Zhu-lin Xie, Aaron Taggart, Karen L. Mulfort, Alex B. F. Martinson, Lin X. Chen, Uta Ruett and David M. Tiede

Experimental Section.

S1. Materials Preparation

S1.1. Substrate preparation

Alkaline aluminoborosilicate glass substrates (Delta Technologies, LTD, CB-1511), ITO/glass electrodes (Delta Technologies, LTD, CB-1511) and silicon substrates (Kunshan Sino Silicon Technology Co., LTD, DAWBDUS002A0240) were cut into 1.5 × 1.5 cm wafers. Before ALD or SIS was performed, the glass wafers were sonicated in detergent/water, water, acetone, and isopropanol 10 min each and blown dry with a N_2 stream. The silicon substrates were prepared likewise but without the detergent.

S1.2. Atomic Layer Deposition

ITO and IZO depositions were carried out in a commercial ALD reactor (Cambridge Nanotech, Inc., Sven 200). The ALD chamber was held at 150 °C for the deposition with a constant N_2 stream at 20 sscm. TMIn and TDMASn were heated to 50 °C and 65 °C, respectively, and the gas delivery manifold to 80 °C. After the samples were brought into the chamber, it was evacuated, and the samples were cleaned with 4 cycles of 30 second pulses of ozone with 20 second purge times. The ozone pulses were split into three ten-second pulses with five seconds purging in between. Trimethylindium (TMIn, Strem, 49-2010, 98%) tetrakis(dimethylamido)tin(IV) (TDMASn, 98-4050, >99.99%), and diethylzinc (DEZ, Sigma-Aldrich, 668729) were used without further purification. The system is outfitted with an O3 corona arc discharge generator (DelOzone) which produces ~5 wt% O_3 in O_2 from ultra-high-purity O_2 (Airgas North Central Inc.) delivered at 0.4 L/min during dosing. The ALD reaction timings followed the sequence t_1 (t_2)– t_3 – t_4 – t_5, where t_1 and t_2 are the exposure times for the TMIn and TDMASn or DEZ, t_3 is the purge time for the metal precursors, and t_4 and t_5 are the exposure and purge times for the ozone pulses. All times are recorded in seconds. For ITO films, the timing was 0.05 (0.015) – 20 – 30 – 20 for 25 supercycles, and TDMASn was pulsed every twentieth cycle starting with the eleventh cycle. For IZO films, the timing was 0.05 (0.03) – 20 – 30 – 20 for 50 supercycles, and DEZ was pulsed every tenth cycle starting with the fifth cycle. The ozone cycles were split into three ten-second pulses with five seconds purging in between. The ellipsometric data were collected by using a J. A. Woollam α-SE in a wavelength range of 500–900 nm at angles of 65°, 70°, and 75°. CompleteEase software was used to

analyze the ellipsometric data. ITO samples were measured to be 49.5 ± 1.1 nm, and IZO samples were 51.9 ± 0.4 nm based on five witness substrates each. Silicon wafers were used for reference films.

After deposition, the samples were annealed in a 1" diameter quartz tube furnace in 3.5% H_2/N_2 forming gas (Airgas North Central Inc.)at 1 atm with a temperature ramp rate of 5 °C/min. Films were held at 250 °C for 1 hour.

S1.3. Sequential Infiltration Synthesis

Sequential infiltration synthesis (SIS) was performed based on a previously reported procedure.(Taggart *et al.*, 2021) 4 wt % poly(methyl methacrylate) (PMMA) (Sigma-Aldrich, 200336-50G, average MW ~ 15000) in toluene solution (Sigma-Aldrich, 244511-100 ML, 99.8%) was prepared. Si with 300 nm thermal oxide (University Wafer 1116) and glass substrates were sonicated in ethanol and then isopropanol for 15 min each, followed by a 5 min UV-ozone treatment (Jelight Company Inc., Model 18) to remove organic contaminants. Substrates were then spincoated with PMMA at 2000 rpm for 30 s in a Laurel Technologies spin coater (WS-400B-6NPP/LITE).

SIS was performed in a commercial ALD reactor (Savannah 200). Trimethylindium (TMIn) (Strem, 49-2010, 98+%) was held at 50 °C, and hydrogen peroxide (Sigma-Aldrich, 30 wt % in water, 216763-100 ML) was held at room temperature. The temperature of the gas delivery manifold and reactor chamber was 80 °C. The entire reaction was performed with a carrier gas of N_2 at 5 sccm. TMIn was dosed for 5 s and exposed to the PMMA films for 30 s. The reactor was then purged for 5 s to remove excess TMIn from the headspace and prevent CVD reactions. Next, H_2O_2 was dosed for 2 s and exposed to the film for 60 s, followed by a 2 min purge.

Samples were annealed in a 1″ diameter quartz tube furnace in ultra-high purity O_2 then 3.5% H_2/N_2 forming gas (Airgas North Central Inc., X02NI96C3000460) at 1 atm with a temperature ramp rate of 7 °C/min. Films were held at the annealing temperature of 300 °C for 1 hr. *Safety note! O_2 and forming gas should never be mixed, especially not at elevated temperatures.*

S2. Scanning Electron Microscopy

SEM measurements were collected on a Hitachi S-4700-II SEM, at working distances of 4 mm with an accelerating voltage of 10 kV.

S3. Grazing-Incidence Total X-ray Scattering (GITXS) Collection and Pair Distribution Function (GIPDF) Analysis

S3.1. Calculating Critical Angle of In_2O_3

Because the films were primarily composed of In_2O_3, the physical parameters of each were estimated to be that of In_2O_3. The critical angle was calculated with Equation 1:

$$(1)\ \alpha_c = \lambda\sqrt{\frac{r_e\rho}{\pi}}$$

where α_c is the critical angle, λ is the wavelength of the incoming photons, r_e is the electron mass, and ρ is the material density.

S3.2. Calculating the Attenuation Depth of In_2O_3

The attenuation depth of the X-rays into the film can be calculated using Equations 2-6:

$$(2)\ \Lambda = \frac{\lambda}{\sqrt{2}\pi} * \left(\sqrt{\left(\alpha_i^2 - \alpha_c^2\right)^2 + 4\beta^2} - \left(\alpha_i^2 - \alpha_c^2\right)\right)^{-\frac{1}{2}}$$

$$(3)\ \beta = \frac{\lambda\pi}{4}\mu_r$$

$$(4)\ \mu_r = \rho * \sum_i P_i mac_i$$

$$(5)\ P_{atom} = w_{atom} / \sum_i w_i$$

$$(6)\ w_{atom} = c_{atom} * m_{atom}$$

where Λ is the penetration depth, α_i is the incident angle, β is the absorption term for the index of refraction, μ_r is the linear absorption coefficient, ρ is the electron density, P is the relative contribution of a particular element to the attenuation, *mac* is the mass attenuation coefficient of each atom, *w* is the summed atomic weight of each atom per unit cell, *c* is the number of atoms per unit cell, and *m* is the atomic weight.

S3.3. GITXS Data Collection

Total scattering data was collected at Beamline 11-IB-D at the Advanced Photon Source at Argonne National Laboratory with a photon energy of 58.6 keV (0.2116 Å) (or 87.6 keV for the SIS-growth In_2O_3 sample). A custom holder consisting of a 10-mm pin, goniometer head, and centering assembly mounted to a Physik Instrumente H-840 hexapod. The specimen is secured to the pin through suction provided by a series of channels running along the height of the pin attached to a vacuum pump. These channels serve a second purpose as alignment pattern (cross) on the face of the pin, used during mounting of the specimen and standard materials (calibrants) to ensure consistent positioning of the samples with respect to the X-ray beam. The point of confusion of the hexapod, assigned to the center of

the top surface of the sample (taking into account the height of the pin and the thickness of the specimen), is aligned to X-rays. The calibration of the measurement is carried out using NIST-traceable SRM 674b CeO_2 material loaded into a Kapton capillary mounted in the center plane of the pin perpendicular to the path of the X-rays using the aforementioned alignment pattern of the pin surface. This approach generates calibration parameters for the center plane of the pin, which remain valid for the measurements of other samples: the footprint of the beam during the measurements on the flat specimen, if aligned correctly, remains centered on that plane. Data has been collected on a PerkinElmer 1621 a-Si detector using 300 1-second exposures summed into one image for a total collection time of 5 minutes per data point. The length of the exposure has been optimized to yield satisfactory signal quality without overexposing the detector. Six of these images are collected per sample. Data has been acquired using the QXRD program; dark current and flat-field corrections have been applied within the same software. Data was collected at the incident angle of 0.048° for In_2O_3 unless stated otherwise.

S3.4. Data Reduction for GIPDF Analysis

The python package pyFAI(Kieffer *et al.*, 2020) was used for data calibration, masking, and averaging of the 2D images. Data from $Q_{xy} < \sim 0.5$ Å^{-1} were masked to avoid specular reflections and the Yoneda peak. Following this, another open-source python package, pygix, was used to perform geometric corrections of the raw data to give images with χ as the azimuthal axis. (Dane 2021) This corrected data was then integrated in 10° slices along χ using pygix, where each slice was centered at a multiple of 10°. Only data above the sample horizon was used because the X-rays were largely blocked by the substrate and holder below $Q_z = 15$ Å^{-1}. Additionally, the quadrant that was occluded by the beam stop was excluded from the data analysis, leaving a single quadrant for analysis.

The data reduction for all samples was performed in the range 0.8 Å^{-1} < Q < 19 Å^{-1} using PDFGetX3, a python package for PDF data reduction.(Juhas *et al.*, 2013)

S4. Bragg Peaks Analysis

To determine the orientation of the two ITO films from the GIWAXS data, the position of the Bragg peaks was correlated to diffraction planes in a known ITO crystal structure.(Nadaud *et al.*, 1998) The VESTA software was used to project the crystal structure and simulate different lattice planes in the structure. The planes of interest were determined by a simulated powder pattern from VESTA. (Momma & Izumi, 2011) The peaks with the highest intensities were projected in the structure. Three strong Bragg peaks at Q = 2.1 Å^{-1}, 2.5 Å^{-1}, and 3.5 Å^{-1} were selected for analysis, which correlated to the Q values for

the first three Bragg peaks in the experimental data. From here, the structure was rotated until the planes were all aligned with the χ positions of the Bragg peaks in the 2D data. Note that, unlike the GIPDF data, when a Bragg peak is along the y axis of the 2D data, the corresponding diffraction plane is *parallel* to the substrate. Likewise, peaks along the x axis correspond to planes *perpendicular* to the substrate.

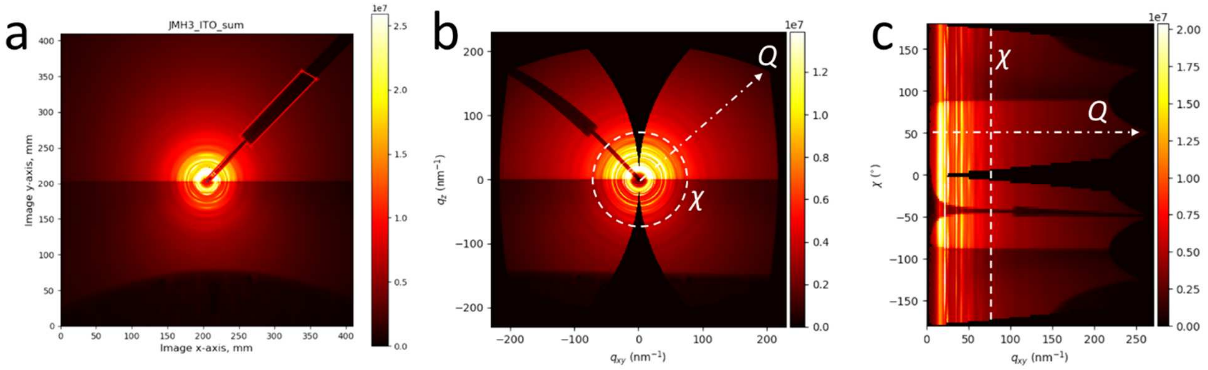

Figure S1 How to generate a pole figure after correcting for the GI geometry. (a) The raw 2D data. (b) The same data after geometric corrections for the GI geometry. The azimuthal axis is now representative of χ, the actual orientation for the sample. Q is the radial axis. (c) A pole figure of the same data showing the Q and χ axes from (b).

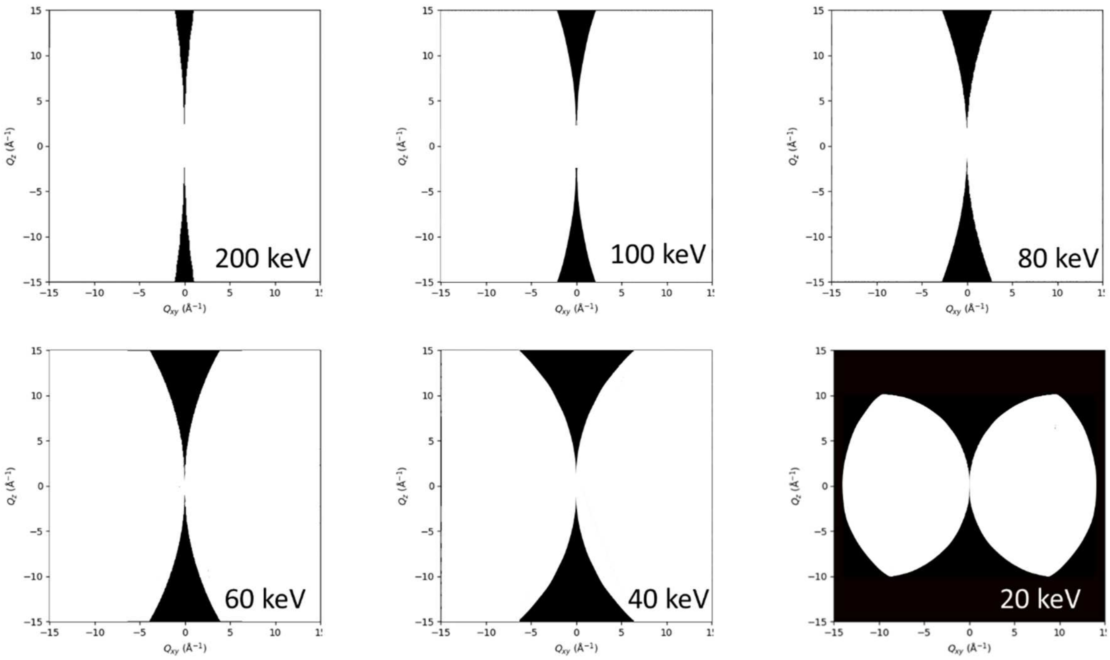

Figure S2 Geometrically corrected 2D data showing the geometric space accessible with different energy X-rays. The areas in white are accessible, and the areas in black are not. While the shape of the distorted 2D data remains the same, the missing wedge is wider at smaller Q values for lower energy X-rays. The images were generated in pygix by setting the detector distance parameter in the program to an arbitrarily small value to generate as large a Q range as possible. Note: No masking is done in these images.

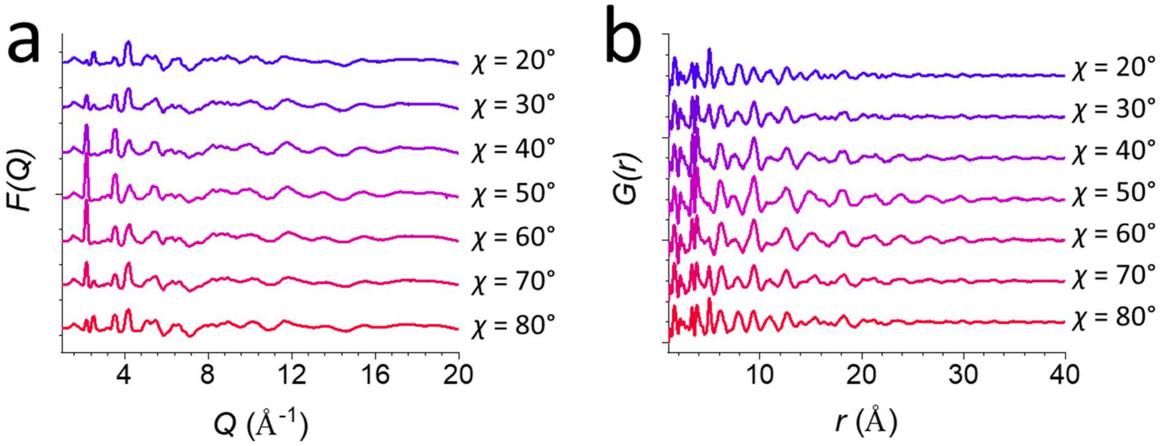

Figure S3 (a) $F(Q,\chi)$ for commercial ITO showing the full Q range used for the Fourier transformation. The angles refer to the χ integration mid-values, with 0° corresponding to features parallel to the supporting substrate and 90° corresponding to features perpendicular to the substrate. (b) The resultant $G(r,\chi)$ from (a) out to r = 20 Å.

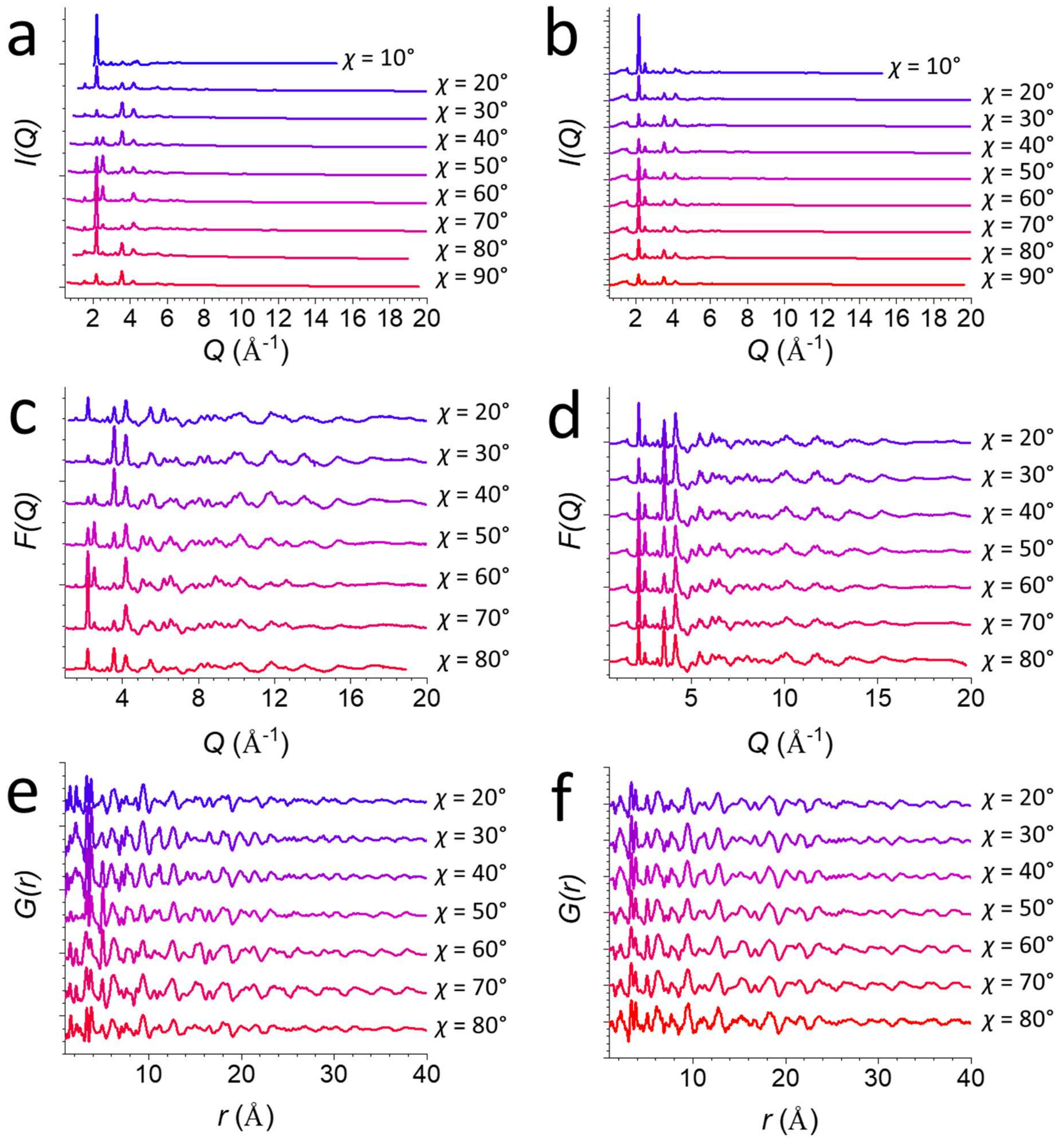

Figure S4 (a) The glass-background-subtracted $I(Q,\chi)$ for ALD-grown ITO films showing the full Q range used for the Fourier transformation. The angles refer to the χ integration mid-values, with 0° corresponding to features parallel to the supporting substrate and 90° corresponding to features perpendicular to the substrate. The film here was measured with α_i = 0.036°. (b) The same as (a), but with $\alpha_i = \alpha_c$ = 0.048° (c,d) The resultant $F(Q,\chi)$'s from (a) and (b). (e,f) The resultant $G(r,\chi)$'s from (c) and (d) out to r = 20 Å. Note that the film measured at 0.048° was not masked around the missing wedge.

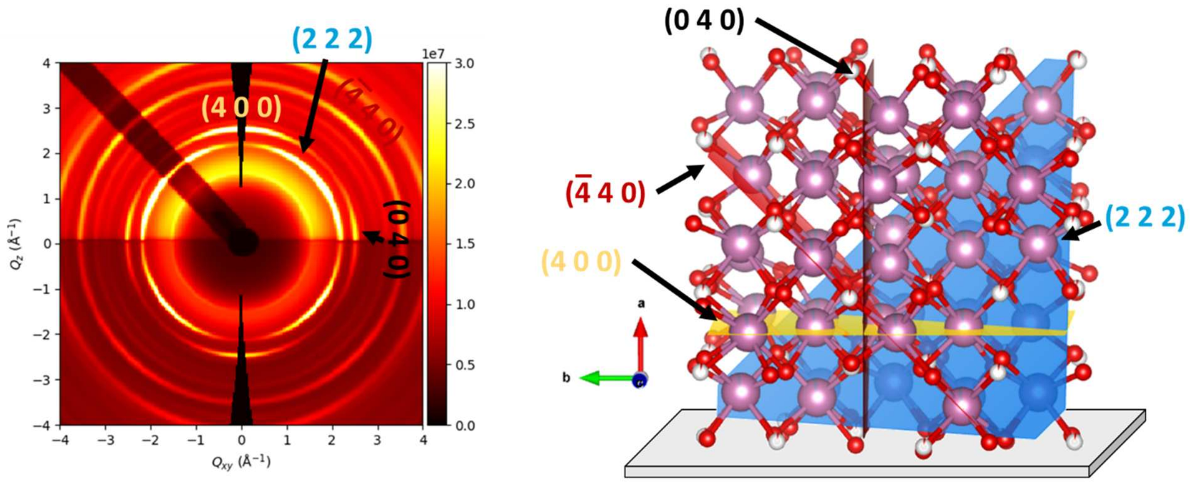

Figure S5 Orientation of a commercial ITO film obtained though the traditional GIWAXS method. On the left shows the geometrically corrected 2D data with the Bragg spots spread out into arcs along χ. The relevant spots are labeled with the Miller indices. On the right is the proposed orientation of the ITO structure with the diffraction planes labeled.

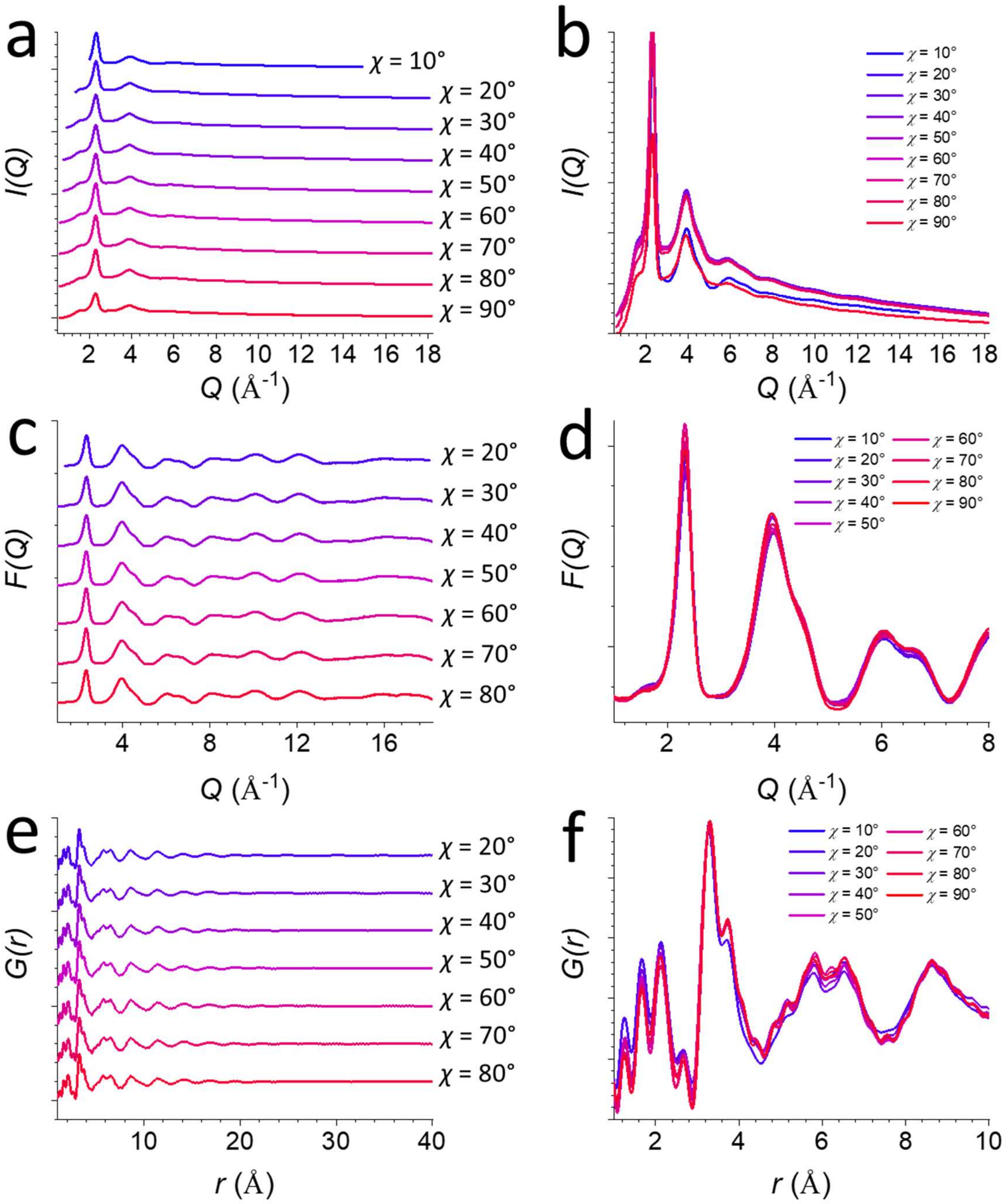

Figure S6 (a,b) The glass-background-subtracted *I(Q,χ)* for ALD-grown IZO films showing the full *Q* range used for the Fourier transformation. The films were measured at $\alpha_i = \alpha_c = 0.047°$. The angles refer to the χ integration mid-values, with 0° corresponding to features parallel to the supporting substrate and 90° corresponding to features perpendicular to the substrate. (c,d) The resultant *F(Q,χ)* from (a,b). (e,f) The resultant *G(r,χ)* from (c,d).

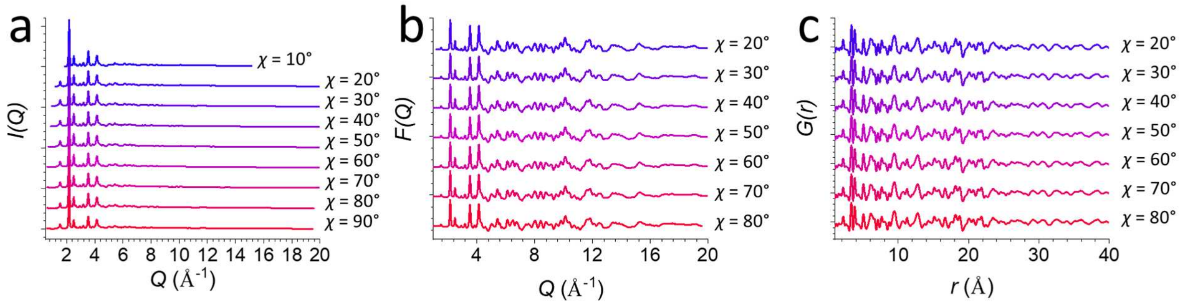

Figure S7 (a) The glass-background-subtracted *I(Q,χ)* for SIS-grown In_2O_3 films showing the full *Q* range used for the Fourier transformation. The films were measured at $\alpha_i = \alpha_c = 0.048°$. The angles refer to the χ integration mid-values, with 0° corresponding to features parallel to the supporting substrate and 90° corresponding to features perpendicular to the substrate. (b) The resultant *F(Q,χ)* from (a). (c) The resultant *G(r,χ)* from (b).

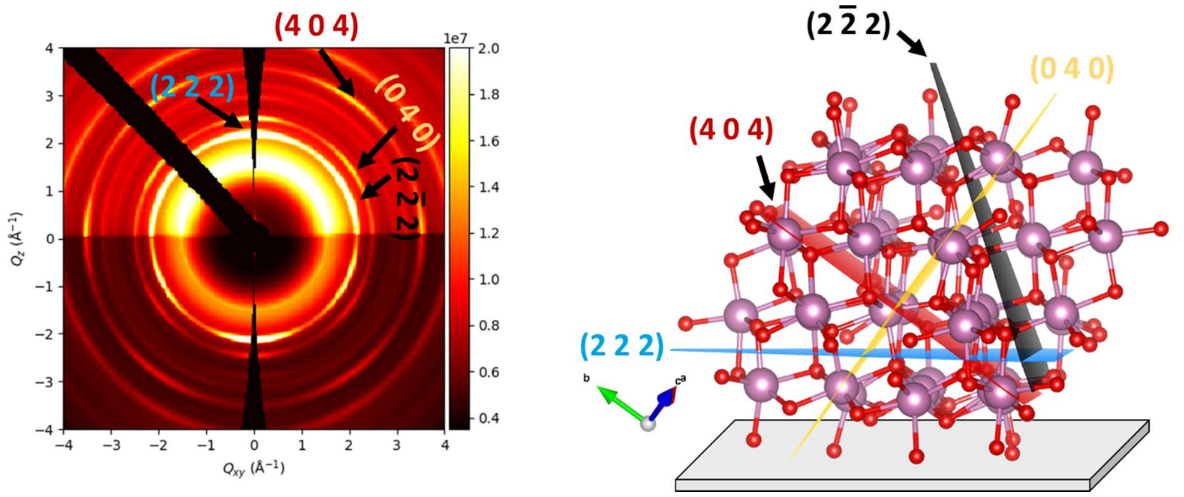

Figure S8 Orientation of an ALD-grown ITO film obtained though the traditional GIWAXS method. On the left shows the geometrically corrected 2D data with the Bragg spots spread out into arcs along *χ*. The relevant spots are labeled with the Miller indices. The (4 0 0)* Bragg spot does not match the expected pattern for this orientation and is likely due to a minority orientation. On the right is the proposed primary orientation of the ITO structure with the diffraction planes labeled.

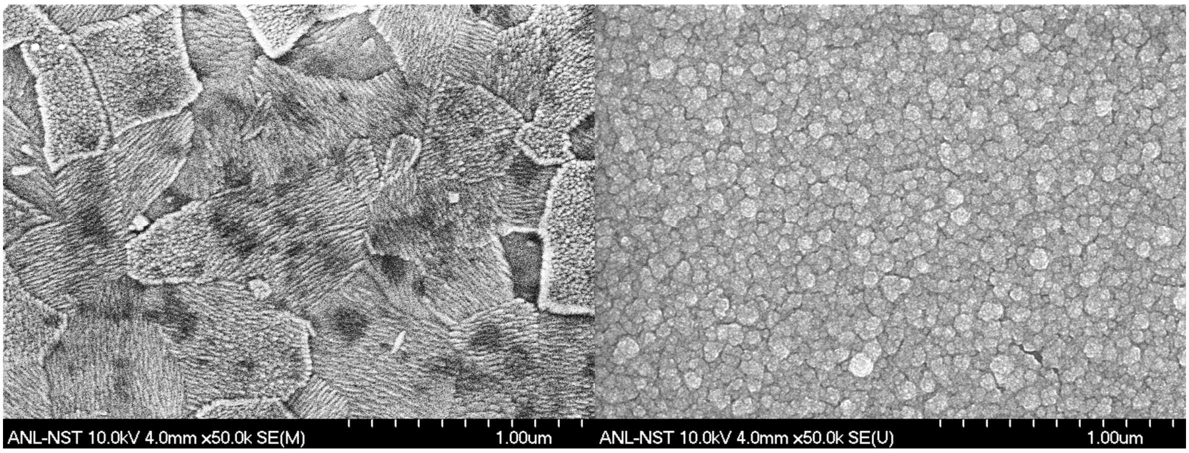

Figure S9 (Left) SEM image of a commercial ITO film. (Right) SEM image of an ALD-grown ITO film.

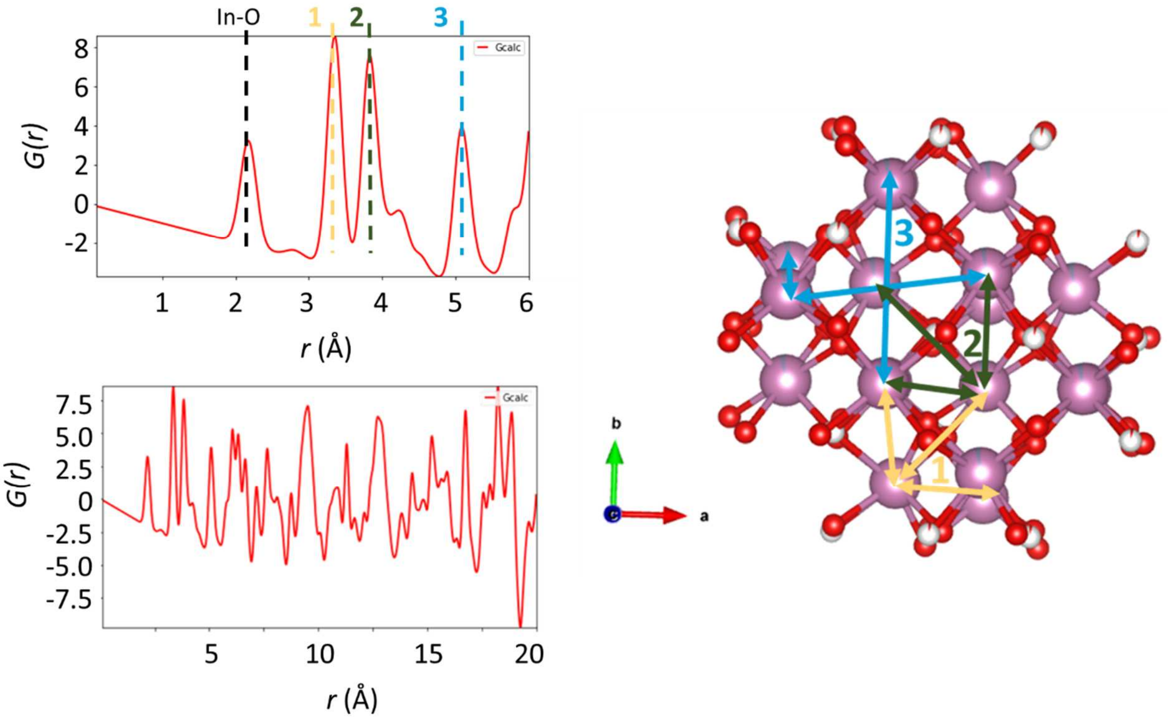

Figure S10 A simulated PDF pattern of In_2O_3 crystal structures, shown with different *r* ranges. The simulations treated the structures isotropically, meaning that any effects from GI are not considered here. On the right is the crystal structure with different pair correlations labeled, with the corresponding peaks labeled on the pattern to the left.

S5. References

Dane, T. & Kieffer, J. (2021). *pygix.*

Juhas, P., Davis, T., Farrow, C. L. & Billinge, S. J. L. (2013). *J. Appl. Crystallogr.* **46**, 560-566.

Kieffer, J., Valls, V., Blanc, N. & Hennig, C. (2020). *J. Synchrotron Rad.* **27**, 558-566.

Momma, K. & Izumi, F. *J. Appl. Crystallogr.* (2011) **44**, 1272-1276.

Nadaud, N., Lequeux, N., Nanot, M., Jové, J. & Roisnel, T. (1998). *J. Solid State Chem.* **135**, 140-148.

Taggart, A. D., Jeon, N., Rozyyev, V., Karapetrova, E., Zaluzec, N. J., Waldman, R. Z., Darling, S. B., Elam, J. W. & Martinson, A. B. F. (2021). *J. Phys. Chem. C* **125**, 21191-21198.
